# Striking the Right Balance Determines TB or Not TB

**DOI:** 10.3389/fimmu.2014.00455

**Published:** 2014-10-08

**Authors:** Somdeb BoseDasgupta, Jean Pieters

**Affiliations:** ^1^Biozentrum, University of Basel, Basel, Switzerland

**Keywords:** *Mycobacterium tuberculosis*, macrophage, immunity, inflammation

## Abstract

*Mycobacterium tuberculosis* continues to be one of the most successful pathogens on earth. Upon inhalation of *M. tuberculosis* by a healthy individual, the host immune system will attempt to eliminate these pathogens using a combination of immune defense strategies. These include the recruitment of macrophages and other phagocytes to the site of infection, production of cytokines that enhance the microbicidal capacity of the macrophages, as well as the activation of distinct subsets of leukocytes that work in concert to fight the infection. However, being as successful as it is, *M. tuberculosis* has evolved numerous strategies to subvert host immunity at virtual every level. As a consequence, one third of the world inhabitants carry *M. tuberculosis*, and tuberculosis continuous to cause disease in more than 8 million people with deadly consequences in well over 1 million patients each year. In this review, we discuss several of the strategies that *M. tuberculosis* employs to circumvent host immunity, as well as describe some of the mechanisms that the host uses to counter such subversive strategies. As for many other infectious diseases, the ultimate outcome is usually defined by the relative strength of the virulence strategies employed by the tubercle bacillus versus the arsenal of immune defense mechanisms of the infected host.

## Introduction

Tuberculosis has affected human beings for centuries. Still, one-third of the world’s population is infected with *Mycobacterium tuberculosis*, of which close to 9 million people progress into active disease while the rest remains asymptomatic (1–4). In recent years, the problem of drug resistant strains of *M. tuberculosis* that further limits the possibilities for therapeutic interventions has only added to the global burden of tuberculosis.

The usual route of infection occurs via aerosols containing mycobacteria. These aerosols are released into the environment through coughing by individuals who suffer from active tuberculosis and are inhaled by recipients into the respiratory tract. There, alveolar macrophages phagocytose the bacilli in an attempt to eliminate the pathogen (5–7). However, *M. tuberculosis*, being an extremely slowly growing microbe, with a generation time of 15–20 h, has evolved elaborate strategies to circumvent the innate antimicrobial response that operates within macrophages, resulting in a long-term cohabitation of *M. tuberculosis* with their host cells. Once inside the host, *M. tuberculosis* can meet several fates: the bacteria can be killed and eliminated, they can survive and proliferate, resulting in active disease, or the bacilli stop growing but reside within the host in a latent state; as a result of the latter, many individuals harbor life-long infections of slowly growing or even dormant bacteria that have also the potential to become reactivated; whether active tuberculosis results from reactivation or *de novo* secondary infections remains unclear in many cases (8–12).

Once within the host, *M. tuberculosis* excels at circumventing macrophage immune defense mechanisms and interferes at different levels with the generation of an effective acquired immune response, largely through the modulation of and interference with cytokine-mediated immune activation mechanisms (1, 13, 14). However, other than the mycobacterial load itself, also the resulting immune activation that aims to control the infection can contribute to the pathology associated with tuberculosis thereby resulting in the development of active disease both at the site of infection, in the lungs, and also within other organs following mycobacterial dissemination (15–17).

Progression of an *M. tuberculosis* infection usually includes an initial innate phase followed by an adaptive immune response that can progress into a chronic state (2, 18–20). In this article, the capacity of mycobacteria to evade the microbicidal activity of macrophages as well as the response of the host through innate and adaptive immune responses will be reviewed, with a focus on those issues that determine whether the balance tips in favor of the pathogen or the host.

## Entry of Mycobacteria into Macrophages

The primary cells in the respiratory tract that are in charge of host defense are the alveolar macrophages, and due to the presence of a diverse number of ligands on the mycobacterial surface, a large repertoire of macrophage receptors can be involved in pathogen recognition and internalization. Such receptors include complement receptors, mannose receptors, scavenger receptors, and C-type lectins such as DC-SIGN, pattern recognition receptors, and surfactant protein receptors. Any of these receptors can lead to uptake through receptor-mediated phagocytosis by recognizing molecules on the mycobacterial surface (21–28). Several lines of evidence implicate the so-called “mammalian cell entry” proteins (Mce 1–4) expressed by mycobacteria in the internalization of mycobacteria into phagocytes (29, 30). Mce proteins contain a well characterized Arg-Gly-Asp (RDG) motif that is responsible for binding to integrins at the mammalian cell surface (31); also, the Mce proteins have been suggested to represent ABC transporters possibly involved in lipid import (32, 33). The precise mode of action of these Mce proteins remains unclear, as *M. tuberculosis* strains lacking Mce1 are hypervirulent in mice whereas disruption of Mce 2-4 results in attenuation (34–36). One other key component regulating mycobacterial entry is plasma membrane resident cholesterol, whose depletion from the macrophage membrane prevents bacterial entry (37). Cholesterol could serve to directly provide entry of mycobacteria via the plasma membrane or allow the stable expression of receptor and/or signaling molecules that assist mycobacterial entry (38, 39).

Another class of molecules, essential for microbial recognition in both macrophages and dendritic cells, are Toll-like receptors (TLRs). The cytoplasmic domain of TLRs is linked to adaptor proteins including not only myeloid differentiation primary response gene (88) product (MyD88) but also MyD88 adapter-like (Mal), TIR (Toll/Il1 receptor)-domain-containing adapter-inducing interferon-β(TRIF) and TLR 4 adaptor protein (TRAM) in case of TLR4. Those adaptors then recruit interleukin-1 receptor-associated kinase (IRAK) 2 and 4, leading to the activation of nuclear factor ‘kappa-light-chain-enhancer’ of activated B-cells (NFkB) (40–42). Mycobacterial peptidoglycans, lipopeptides, and mycobacterial DNA are recognized by TLR2, 4, and 9, respectively (43). TLR activation has numerous consequences, including the production of inflammatory mediators, the regulation of phagocytosis, as well as connecting the phagosomal pathway with autophagosomes thereby inducing rapid mycobacterial killing (44–49). Whereas a functional connection between phagocytosis and autophagosome formation has been mainly demonstrated using model particles (48). It has also been shown that macrophages harboring *M. tuberculosis* eliminate the pathogen via autophagy (50). One additional advantage of TLR-mediated activation of phagocytosis and autophagosome formation may be a more efficient regulation of antigen processing and presentation by infected macrophages (47). Also, in human macrophages, TLR activation was shown to result in upregulation of a vitamin D-induced antimicrobial pathway that inhibits the growth of *M. tuberculosis* (51, 52).

## Balancing Survival Strategies Employed by *M. tuberculosis* Against Host Defense Strategies

Following receptor recognition and phagocytosis of *M. tuberculosis* by alveolar macrophages, a plethora of reactions are initiated by both the host and the mycobacteria, which together determine the outcome of the infection. In general, macrophages tend to destroy anything that is phagocytosed; however, pathogenic mycobacteria have developed an elaborate range of strategies to combat the bactericidal milieu encountered upon ingestion.

### Blocking the delivery of *M. tuberculosis* to lysosomes

Several host factors are involved in the modulation of intracellular trafficking of mycobacteria within macrophages. One of these is the protein coronin 1 (also known as P57 or TACO, for tryptophan aspartate containing coat protein) that is present at the cell cortex of macrophages, and is actively recruited and retained at the cytosolic face of nascent mycobacteria-containing phagosomes (7, 8, 37, 53). Coronin 1-dependent blockage of phagosome maturation occurs through activation of the Ca2+/calcineurin pathway, possibly involving dephosphorylation of molecules normally involved in the regulation of intracellular trafficking of phagosomal cargo (54–59). The exact mechanisms of coronin 1 retention at the mycobacterial phagosome remain to be determined and may involve the enzyme lipoamide dehydrogenase that is secreted by mycobacteria and is also known to be involved in resisting the toxic effects of nitrogen intermediates generated by host cells (60, 61). In line with an important role for calcineurin in promoting mycobacterial survival, it was shown that calcineurin inhibitors such as cyclosporin A and FK506 can induce mycobacterial transfer to lysosomes and their killing (54, 55, 62). Whether or not blocking of this pathway may be useful for the treatment of mycobacterial infections is unclear, since both calcineurin and coronin 1 are important molecules involved in T-cell homeostasis (56, 59, 63, 64).

Mycobacteria-containing phagosomes are known to continuously shed off phosphatidylinositol-3-phosphate (PI3P), a key phosphoinositide known to regulate the phagosomal acquisition of lysosomal constituents (65). Also, dephosphorylation of the vacuolar protein sorting protein 33B prevents membrane fusion events that are crucial for phagosome maturation (66). It is quite possible that different host factors are being hijacked at distinct phases of the infection process, and future work may shed further light on the precise kinetics of the above mentioned processes.

### Role of mycobacterial virulence factors in preventing phagosome–lysosome fusion

In the course of the co-evolution with their host cell, the macrophage, *M. tuberculosis* has built up a large arsenal of virulence factors that can interfere with host immune functions at different levels. The virulence repertoire of mycobacteria comprises distinct kinases, phosphatases, metalloproteinases, and pore forming proteins, which together constitute a potent infectious challenge.

One of these virulence factors is the exported repeated protein (Erp) that is required for intracellular replication of pathogenic mycobacteria (67). Erp proteins have three domains, where the amino terminus harbors a signal sequence, a central variable domain containing PGLTS repeats, and a proline–alanine rich C-terminus. Erp is important to maintain the cell wall integrity of mycobacteria through the riboflavin metabolism pathway and is required for its survival both in macrophages and *in vivo* (68, 69).

Another mycobacterial factor, the eukaryote-like serine/threonine protein kinase protein kinase G (PknG), is released by mycobacteria into the host cytosol, thereby blocking phagosome–lysosome fusion (39, 70, 71). As a consequence, mycobacteria lacking PknG are rapidly transferred to lysosomes and degraded (70, 72–75). Interestingly, although highly homologous to eukaryotic serine/threonine protein kinases, PknG contains a binding pocket for substrates that is shaped by a unique set of amino acid side chains that are not found in any human kinase. Therefore, PknG is well amenable to specific inhibition without the risk of targeting host kinases, thereby possibly allowing the inhibition of intracellular replication of pathogenic mycobacteria without adverse effects on host cells (73, 76, 77).

Within macrophages, mycobacteria also release a phosphatase, called secreted acid phosphatase of *M. tuberculosis* (SapM), that hydrolyzes phosphatidyl inositol 3 phosphate (PI3P), which, as mentioned above, is involved in regulating phagosome–lysosome fusion. As a consequence of depleting PI3P locally, mycobacterial SapM contributes to the inhibition of phagosome maturation and thereby intracellular mycobacterial survival (78, 79). Furthermore, a putative zinc-dependent metalloprotease (Zmp1) released by mycobacteria interferes with phagolysosome biogenesis by interfering with caspase 1-dependent activation. Activated caspase 1 is known to cleave pro-interleukin-1β in order to generate active interleukin-1β and activating the inflammasome complex (80, 81). The absence of Zmp1 suppresses this activation and thereby promotes phagosome–lysosome fusion and elimination of mycobacteria (82). Also, the enhanced intracellular survival (Eis) protein is known to prevent natural killer (NK) cell-dependent reactive oxygen species (ROS) generation via its aminoglycosyl aminotransferase activity (83). Eis protein dampens tumor necrosis factor-α (TNFα) and interleukin-10 production thereby preventing macrophage activation. Macrophage activation through TNFα or interferon-γ results in phagosome–lysosome fusion. Eis protein inhibits the generation of ROS in the cell and thereby prevents the onset of macrophage activation and autophagy (84).

Finally, although not directly involved in the modulation of phagosome–lysosome fusion, the type VII secretion system encoded by the RD1 locus is known to produce and secrete the early secretory antigenic target (ESAT) 6 and culture filtrate protein (CFP) 10, which are key components for promoting cytosolic escape of mycobacteria (85–88). The ESX-1 gene cluster exhibits higher expression when mycobacteria encounter a reduced pH, and perhaps the ESX-1 system is therefore a mechanism allowing the cytosolic escape of those mycobacteria that may have been transferred to the lysosome (89–92).

### Macrophage activation as a mechanism to counteract mycobacterial infections

One of the consequences of infection is the activation of innate immune responses through the release of macrophage-activating molecules such as various cytokines including interferon-γ (93–97). Activated macrophages then modulate intracellular trafficking of mycobacteria and promote phagolysosome formation resulting in mycobacterial destruction (98, 99). Macrophage activation allows the replenishment of phosphoinositides at mycobacteria-containing vesicles and thereby promotes the exchange of Rab5 by Rab7, which is crucial to its fusion with the lysosome (79). Also, the natural resistance-associated macrophage protein1 (Nramp1), a metal ion transporter that scavenges metal ions from the microbe-containing vesicles (100–103), is modulated by macrophage-activating cytokines such as interferon-γ (104). Defects in Nramp production lead to susceptibility to mycobacterial infections (103, 105, 106).

A recent study shows that inflammatory stimuli such as induced by interferon-γ or TNFα result in the phosphorylation of the protein coronin 1, which as described above is crucial to allow mycobacterial survival in non-activated macrophages. Activation-induced phosphorylation of coronin 1 results in its delocalization from the cell cortex to cytoplasmic punctae, thereby reprograming the macrophage endocytic pathway from receptor-mediated phagocytosis to macropinocytosis (107). Interestingly, relocated coronin 1 activates phosphatidylinositol (PI)-3-kinase, which is required for an early burst of PIP3 that is known to be essential for membrane ruffle formation and induction of macropinocytosis (107–110).

Macropinocytic uptake of mycobacteria along with the upregulation of the interferon-γ response genes plays a critical role in pathogen elimination (111–113). These interferon-γ-induced genes include the LPS-stimulated RAW 264.7 macrophage protein 47 homolog (LRG-47), also known as Irgm1 (114–116), as well as a series of guanylate-binding proteins (GBPs) that are involved in the activation of diverse host defense mechanisms, including activation of the phagocyte oxidase, generation of antimicrobial peptides, and induction of autophagy (117, 118). Interestingly, recent work suggests that Irgm1/LRG-47 fails to associate with mycobacterial phagosomes (119) and that deficiency of Irgm1/LRG-47 results in an interferon-γ-dependent collapse of the lymphomyeloid system, causing general susceptibility to pathogens (120–122). Thus, these immunity-related GTPases may have a rather broad function in shaping the immune system as also exemplified by recent work implicating Irgm1/LRG-47 in hematopoietic stem cell proliferation (116, 123).

### Key host defense factors involved in the elimination of mycobacteria upon macrophage activation

Once in the phagosome, a number of antibacterial molecules directly threaten mycobacterial viability. Among these, reactive oxygen intermediates in the form of hydrogen peroxide are generated by macrophages via the oxidative burst, and effectively limits mycobacterial growth within macrophages (124, 125). Interestingly, vitamin C along with the antibiotic isoniazid was recently shown to effectively kill extreme and total drug resistant *M. tuberculosis* through the induction of ROS (126).

Reactive nitrogen intermediates are generated by cytokine-activated macrophages through inducible nitric oxide synthetase using l-arginine as the substrate (127, 128). Upon mycobacterial infection, mycobacteria upregulate macrophage arginase, which then catalyzes l-arginine to l-ornithine and urea and thereby prevents nitric oxide generation (129). In addition, the mycobacterial proteasome is also involved in resistance against nitric oxide stress (130, 131), and specific inhibition of mycobacterial proteasomes may be useful to prevent mycobacterial growth within macrophages (132).

As mentioned above, induction of autophagy that is involved in the clearance of many intracellular pathogens is also used by infected host cells to eliminate *M. tuberculosis* (133, 134). Shuttling of *M. tuberculosis* into autophagosomes can occur via a ubiquitin-mediated pathway; recent work has implicated a ubiquitin ligase termed parkin, mutations of which are associated with increased risks for Parkinson disease, in the transfer of *M. tuberculosis* as well as other pathogens to the autophagic pathway (135).

### Formation of granulomas

The survival strategies of mycobacteria and the innate and adaptive immune response elicited to restrict mycobacterial survival and growth come together in a dynamic combat zone called the granuloma (8, 136). Within granulomas, the host attempts to control mycobacterial dissemination, while at the same time propagation of mycobacteria can occur (107, 137–139). Within granulomas, macrophages harboring viable bacteria are being surrounded by a layer of activated macrophages that ensure mycobacterial elimination. These activated macrophages can process and present mycobacterial antigens to an outer layer of T lymphocytes upon which these T-cells secrete cytokines and chemokines that keeps the macrophages in an activated state (5, 13, 20). Thus, granuloma formation represents a delicate balance that is easily disturbed either by enhanced virulence of the mycobacteria or a deteriorating host immunity, such as under immunocompromised conditions (140). New research into the biology of granulomas using different model systems may allow a better understanding of these important structures involved in balancing the host versus the pathogen. TNF-α is essential for the formation and maintenance of granulomas, promoting the release of chemokines, cytokines, and adhesion molecules, which in turn activates and recruit neutrophils required for enhancing the microbicidal activity of macrophages. As a result, anti-TNF-α therapy for the treatment of inflammatory conditions can result in the reactivation of granulomatous infections by inhibiting microbicidal activity (141, 142).

### Cellular immune response against mycobacteria – role of leukocyte subsets and cytokines

Phagocytes play a key role in initiating and directing adaptive immune responses through antigen presentation and expression of co-stimulatory signals and cytokines. However, other leukocyte subsets are also important in the host defense against *M. tuberculosis* (9, 143). These other components of innate immunity include neutrophils as well as NK cells. Neutrophils are not only abundant but also the first cells to arrive at the replicating mycobacteria loci and play an important role in controlling the bacterial load, yet at the same time may be involved in the induction of tissue damage (144–146). NK cells are known to directly lyse the mycobacteria or mycobacteria-harboring macrophages (147). At initial stages of infection NK cells can induce macrophage activation resulting in mycobacterial killing as well as induce macrophage apoptosis also resulting in the destruction of the intracellularly residing bacteria (148, 149).

Since mycobacteria reside in vacuoles within macrophages, major histocompatibility complex (MHC) class II presentation of mycobacterial antigens to CD4+ T-cells is an obvious outcome (1, 150–152); indeed, depletion of CD4+ T-cells from the periphery results in acute mycobacterial pathogenesis (153–155). However, the presence of a pool of *M. tuberculosis* in the cytosol of infected macrophages as well as MHC class I-mediated antigen presentation from phagosomes may also result in the activation of CD8+ T-cells via MHC class I-mediated presentation (91, 156–159). Furthermore, CD1- and MHC class I-restricted CD8+ T-cells can induce perforin/granulysin-mediated lysis of mycobacteria within infected macrophages and dendritic cells (160). Finally, the so-called γ/δ T-cells that represent a small subset of T-cells expressing a γ- and δ-chain instead of the conventional α- and β-chain of the T-cell receptor (161–163) are also important for the control of *M. tuberculosis* within an infected host (164). These γ/δ T-cells appear to recognize specific mycobacterial protein and peptide antigens in an MHC-unrestricted manner (164–168). The precise contribution of γδ T-cells to mycobacterial immunity is still unclear, but given the importance of mycobacterial lipids in the virulence of infection these cells might in fact constitute an important arm of the immune response.

An important function of CD4+ T-cells is the production of cytokines in order to activate macrophages, that in turn upregulate both the capacity to internalize and destroy mycobacteria (107, 153, 169). The predominant pro-inflammatory cytokines that are crucial to counteract mycobacterial infection include interferon-γ, TNFα, interleukin-2, and interleukin-12. Mainly, CD4+ T-cells, and also CD8+ T-cells, NK cells, and infected macrophages all produce interferon-γ, which results in the augmentation of antigen processing and presentation (111, 170, 171). Additionally, cytokine activation increases mycobacterial uptake and elimination through upregulation of macropinocytosis and autophagosome formation (107, 133, 134, 172). The central role for interferon-γ in the control of mycobacterial infections is also illustrated by the high susceptibility of both mice and human beings bearing mutations in genes coding for the interferon-γ receptor (173–175).

Another important cytokine involved in mycobacterial control is TNFα, which is secreted mainly by macrophages and dendritic cells. Similar to interferon-γ, mice lacking TNFα or its receptor are susceptible to mycobacterial infection (176, 177). TNFα plays an important role in granuloma formation and parasite dissemination from the granuloma (178).

The cytokine interleukin-2 that is essential for peripheral T-cell survival, alone or in synergy with other cytokines, is also important to control mycobacterial infection (179, 180). Also, phagocytosis of mycobacteria triggers the production of interleukin-12, which results in the induction of interferon-γ in an autocrine and paracrine fashion (181–185). Furthermore, in macrophages, the cytokine interleukin-4 induces a so-called alternative activation of macrophages, resulting in the upregulation of arginase 1 and suppressor of cytokine signaling (SOCS) 3 like proteins (186). Increased expression of interleukin-4 can result in reactivation of latent tuberculosis (187, 188) illustrating the complex role of interleukin-4 during mycobacterial infections.

Recognition of the mycobacterial cell wall component lipoarabinomannan by macrophages as well as its phagocytosis results in the secretion of the anti-inflammatory cytokine, interleukin-10 (189) which, in turn, dampens the expression of pro-inflammatory cytokines interferon-γ, TNFα, and interleukin-12 (190–194). Hence, interleukin-10 interferes with the host defense against tuberculosis, which is in line with the observed lower bacterial burden in mice lacking interleukin-10 (195). Lipoarabinomannan is also known to induce transforming growth factor (TGF)-β production by macrophage and dendritic cells, thereby counteracting macrophage activation as well as protective immunity against tuberculosis (189, 196–198). These various anti-inflammatory cytokines are expressed to counterbalance the activity of pro-inflammatory cytokines during mycobacterial infection; however, the relative role of pro- and anti-inflammatory cytokines in the disease process remains unclear, and may change depending on either the time course or the precise location of the infection (199, 200).

## Concluding Remarks

The response of the host to mycobacterial infection is complex and multifactorial involving many different immune components. Mycobacteria excel at subverting immune responses through a variety of mechanisms both by involving mycobacterial virulence factors and by hijacking host defense mechanisms. The immune response of the host, resulting in macrophage activation, alters the functionality of these host factors in order to enable pathogen elimination. The continuous tug of war between invading mycobacteria and host immunity finally determines the disease outcome. Future studies combining present day technical advances with the study of suitable host models may help to further unravel the intricate virulence mechanisms of this immensely successful pathogen.

## Conflict of Interest Statement

The authors declare that the research was conducted in the absence of any commercial or financial relationships that could be construed as a potential conflict of interest.
